# Recent Research Progress in China on *Haemonchus contortus*

**DOI:** 10.3389/fmicb.2017.01509

**Published:** 2017-08-24

**Authors:** Chunqun Wang, Fangfang Li, Zongze Zhang, Xin Yang, Awais A. Ahmad, Xiangrui Li, Aifang Du, Min Hu

**Affiliations:** ^1^State Key Laboratory of Agricultural Microbiology, College of Veterinary Medicine, Huazhong Agricultural University Wuhan, China; ^2^College of Veterinary Medicine, Nanjing Agricultural University Nanjing, China; ^3^College of Animal Sciences, Zhejiang Provincial Key Laboratory of Preventive Veterinary Medicine, Zhejiang University Hangzhou, China

**Keywords:** *Haemonchus contortus*, population genetics, anthelmintic resistance, diapause-related genes, aminopeptidase H11, immuno-regulation

## Abstract

*Haemonchus contortus* is one of the most important parasites of ruminants with worldwide distribution that can bring huge economic losses to the breeding industry of cattle, sheep, and goats. In recent 20 years, studies on *H. contortus* in China mainly focused on the epidemiology, population genetics, anthelmintic resistance, structural and functional studies of important genes regulating the development of this parasite, interaction between parasite molecules and host cells and vaccine development against haemonchosis, and achieved good progress. However, there is no systematic review about the studies by Chinese researchers on *H. contortus* in China. The purpose of this review is to bring together the findings from the studies on *H. contortus* in China in order to obtain the knowledge gained from the recent studies in China and provide foundation for identifying future research directions to establish novel diagnostic methods, discover new drug targets and vaccine candidates for use in preventing and controlling *H. contortus* in China.

## Introduction

*Haemonchus contortus* is one of the most economically important parasites infecting small ruminants worldwide. It is a blood-sucking nematode that feeds on blood from capillaries in the abomasum of ruminants, especially for cattle, sheep, and goats (Hoberg and Zarlenga, 2016). Infections with the nematode can cause anemia, weight loss, or even deaths in severely affected animals. In China, *H. contortus* is ubiquitously distributed in the whole country with variable prevalence among different provinces (Wang et al., 2006; Liu et al., 2009; Ma et al., 2014; Yang et al., 2016).

Current control strategies against *H. contortus* primarily rely on repeated anthelmintic treatments. However, the widespread use of anthelmintic drugs has resulted in serious drug resistance problems worldwide in domestic animals (Kaplan, 2004; Kaplan and Vidyashankar, 2012; Kotze et al., 2014). For example, benzimidazole and ivermectin have been used heavily to control nematode infections in China, resulting in the development of drug resistance (Cai et al., 2007; Zhao et al., 2010). Therefore, the emergence of anthelmintic resistance in *H. contortus* necessitates development of new intervention strategies. One of the possibilities is the rational strategy of discovering new anti-parasite drugs and vaccines, built on the deep understanding of the key molecules in the processes of development and reproduction. For *H. contortus*, clear insights into the biological processes at the molecular level might identify key molecules as new drug targets (Britton et al., 2016). In addition, development of immunological control against livestock nematode infections is urgently needed. Significant protection against *H. contortus* has been achieved following vaccination with native protein extracts, demonstrating that vaccination is feasible (Newton and Meeusen, 2003; Mohandas et al., 2016b; Nisbet et al., 2016). In China, the promising vaccine candidates from native protein extracts have been studied, providing some insights in the development of commercial vaccines against *H. contortus* (Yan and Li, 2006; Zhao et al., 2012; Zhou et al., 2014).

This paper reviews research progress in China on *H. contortus*. Particular areas of importance include epidemiological investigation, population genetics, and detection of anthelmintic resistance by applying conventional approaches and molecular methods, structural and functional studies on key molecules in signaling pathways regulating development, interaction between parasites and host cells and vaccine development. The proposed formulation of these views is conducive to identify areas for future research and expedite possibilities for new or updated control measures.

## Survey of *Haemonchus contortus* of Cattle, Sheep, and Goats in China

*Haemonchus contortus* is a blood-feeding nematode infecting small ruminants (Taylor et al., 2016). Animals infected with *H. contortus* can show a series of symptoms, including anemia, emaciation, diarrhea or even death under heavy burden. Damages caused by *H. contortus* can lead to billions of economic losses to the breeding industry (He et al., 2011; Roeber et al., 2013a; Emery et al., 2016), especially for young animals. What’s more, arrested larvae of *H. contortus* are related to the spring rise and can lead to a mass of deaths (Blitz and Gibbs, 1971). China is a large and civilized country with a long traditional history of 5000 years and is also a traditional agricultural country. It locates in Asia, east of the world. Rich vegetation and water resources make it suitable for the development of livestock breeding industry, including cattle, sheep, and goats. In recent 20 years, with the improvement of people’s living standard and increasing supports from the government (e.g., Luo et al., 2005; Ni et al., 2007), more and more farmers turn to breed cattle, sheep, and goats, so the population of cattle, sheep, and goats has been over 300 million since 2008^1^. A large number of cattle, sheep, and goats also provide a suitable environment for the development and spread of bacteria, virus, and parasites, including *H. contortus* which is an important parasitic nematode that can lead to considerable economic losses to the breeding industry of cattle, sheep, and goats. Understanding the epidemiology of *H. contortus* is essential for preventing and controlling of this species.

Until now, there are about 170 Chinese publications (Data based on literature search at CNKI) and five English publications (Data based on literature search at Google Scholar) reported the investigation of *H. contortus* infection of cattle, sheep, and goats in most provinces in China, except for Hainan, Hong kong, Macao, and Taiwan. To understand *H. contortus* infection in ruminant in China, post-mortem diagnosis was the most common method used in the investigations (e.g., Chen et al., 2000; Wang et al., 2005; Yang, 2005), and DNA-based methods were also applied in recent years (Yang et al., 2016, 2017). From the reported data, *H. contortus* is a dominant or key species in most goat farms (e.g., Wei et al., 2013; Zhao et al., 2016; Jiang et al., 2017), and the infection rate ranges from 28 to 100% in the investigated goat farms (e.g., Cao et al., 1995; Chen et al., 2000; Wang et al., 2005, 2006; Yang, 2005; Ma et al., 2014; Yang et al., 2016), and from 0 to 92% in the investigated sheep farms (e.g., Han et al., 1984; Wang et al., 2006, 2014c; Xin, 2010), and from 0 to 61.8% in the investigated cattle farms (e.g., Li et al., 2002; Liu et al., 2009). Surveys on the *H. contortus* infection of both cattle and goats in areas of Chongqing (Li et al., 2001), Guizhou (Yang et al., 2014) and Yunnan (Li et al., 2002) indicated the prevalence of *H. contortus* infection in goats is higher than that in cattle in these investigated areas. However, surveys in Hebei (Yang and Wang, 1984) indicated the prevalence of *H. contortus* infection in sheep was lower than that in cattle. Most of the investigated animals were 0.5- to 4-years-old, but no comparison on the effect of age on the infection of *H. contortus* was made in all the investigations. What’s more, the convenience of trans-regional transport of cattle, sheep, and goats accelerated the spread as well as anthelmintic resistance of *H. contortus* (Li et al., 2007).

To further understand the occurrence and infection patterns of *H. contortus*, Qin et al. (2003) reported the seasonal incidence of predominant gastrointestinal nematodes of sheep in Bashang Altiplano Area, Hebei province. Bashang Altiplano refers to the Altiplano area at 400–1300 m altitude laying in the most north of Hebei province where winter is long and cold and January is the coldest month with the average temperature ranging from -11 to -19°C whereas July is the hottest month with the average temperature ranging from 18 to 20°C, and the annual precipitation is 350–400 mm. The results indicated there were two peak infection periods (March to June; September to January) for *H. contortus* within a year, and the seasonal dynamics of *H. contortus* had obvious spring rise phenomenon. Then, Song et al. (2007) reported the seasonal incidence of egg output and worm burden of *H. contortus* adult of sheep in Songhuajiang area, Heilongjiang province, China, and found egg output and worm burden of *H. contortus* adults reached two peaks within a year. Egg output and worm-burden increased from late April to late May and reached a peak at late May, and then they decreased immediately until late July and reached the second peak at early August and decreased again, and then kept at a low level till to next April. Winter in Songhuajiang area is long and cold, and summer is hot and rainy when July is the hottest month with the average temperature ranging from 20 to 25°C whereas January is the coldest month with the average temperature below -20°C, and the annual precipitation is about 500 mm. Obviously, warm weather in summer would promote the development of *H. contortus* in these two areas, and cold weather in winter would inhibit the development, thus leading to the seasonal dynamics. No data reflecting the peak of transmission in these two areas were related with calving season. Sharing similar climatic characteristics, the seasonal dynamics of *H. contortus* in both areas are alike, however, the infection peak of *H. contortus* in Songhuajiang area was 1 month later in winter than that in Bashang Altiplano area due to colder weather in January, but 1 month earlier in summer than that in Bashang Altiplano area due to warmer weather in July. Knowledge of the infection and seasonal dynamics for *H. contortus* can help us choose the optimum time for effective deworming and decrease the cost.

## Population Genetics of *Haemonchus contortus* in China

Knowledge of genetic variation of *H. contortus* can provide a foundation for understanding the spread of anthelmintic resistance alleles and making the strategy of control of haemonchosis. Until now, three studies have been conducted to explore population genetics of *H. contortus* in China using mitochondrial nicotine amide dehydrogenase subunit 4 (*nad4*) gene, microsatellite markers and the isotype-1 β-tubulin gene (Yin et al., 2013, 2016; Zhang et al., 2016), respectively.

Yin et al. (2013) amplified *nad4* gene from 152 *H. contortus* from sheep and goats from seven different regions in China and analyzed population genetic diversities. As a result, 142 haplotypes of *nad4* gene were identified. The nucleotide diversities and haplotype diversities were 0.0178–0.0369 and 0.993–1.000, respectively, similar with previous reports from different countries. Population genetics analysis revealed that high nucleotide variation (92.4%) was partitioned within population. They concluded that high variations within population, low genetic differentiation and high gene flow among different populations were present in *H. contortus* in China. Yin et al. (2016) then used eight different microsatellite makers to study the genetic variation within and among *H. contortus* populations including 184 adult male worms from seven distinct populations from sheep and goats in China. They found that all eight microsatellite markers were highly polymorphic with high heterozygosity and inbreeding coefficient (*F*_IS_). Moreover, various analysis including AMOVA, F_ST_, phylogenetic, structure, mantel test and population dynamics revealed high within-population variation, low population genetic differentiation and high gene flow for *H. contortus* in China. In addition, Zhang et al. (2016) used the isotype-1 β-tubulin gene to explore the population genetics of *H. contortus* in China. In this study, 132 of *H. contortus* sequences of isotype-1 β-tubulin gene from eight different populations were analyzed. Finally, high haplotype diversities (0.455–0.939) and nucleotide diversities (0.018–0.039) were calculated within each population. The pairwise F_ST_ and AMOVA analysis revealed that high gene flow and low genetic differentiation were present among populations. From the above studies, although three different genetic markers were used, a fairly similar picture of population genetic structure of *H. contortus* in China was identified.

## Anthelmintic Resistance in *Haemonchus contortus* in China

The main method for controlling parasitic nematodes including *Haemonchus contortus* is based on the application of anthelmintics, however, long-term and unreasonable usage of anthelmintics has led to the emergency and development of anthelmintic resistance. Anthelmintic resistance in *H. contortus* has been studied and reported in many parts of the world. A brief review was published by Kaplan and Vidyashankar (2012) reporting the existence of anthelmintic resistance in many countries, including United States, Brazil, South Africa, Australia, New Zealand, and European countries. Nevertheless, up to now, there is no review about anthelmintic resistance in *H. contortus* in China. Over the last two decades, 15 studies have been conducted to detect anthelmintic resistance in parasitic nematodes in the fields using fecal egg count reduction test (FECRT) and (or) egg hatch assay (EHA). However, only five reports focused on or referred to *H. contortus*. In addition to conventional *in vivo* and *in vitro* methods used in those five reports, molecular methods were also utilized to detect benzimidazole resistance in four studies and one study also explored candidate genes for ivermectin resistance in *H. contortus* using a new genome-wide SNP analysis. Hence, we reviewed the research results on anthelmintic resistance in *H. contortus* in China using *in vivo* test, *in vitro* test and molecular test to detect benzimidazole (BZ) resistance and single nucleotide polymorphism (SNP) analysis in ivermectin (IVM) resistant *H. contortus*.

### *In Vivo* Test

A study on the survey of *H. contortus* infection and treatment with albendazole, fenbendazole and ivermectin conducted on a dairy goat farm (400 goats) in Shaanxi province reported that the infection of *H. contortus* were all above 80% and resistance in *H. contortus* to albendazole and ivermectin were detected in this goat farm, however, no resistance was present to fenbendazole. In this study, the routine dose rate (5 mg/kg) of albendazole yielded a FECR of 23.72% and even the three times of the recommended dose rate (15 mg/kg) only reached a FECR of 75.68%. Meanwhile, ivermectin was not effective on this farm, with FECR of 52.29% for the recommended dose rate (0.2 mg/kg) and FECR of 89% for two times of the recommended dose rate (0.4 mg/kg) (Feng et al., 2016). In another study conducted on eight goat farms in Fujian province, albendazole, levamisole, ivermectin and a combination of albendazole and ivermectin were used to test the efficacy against *H. contortus*. The results showed that levamisole, ivermectin and a combination of albendazole and ivermectin were effective on treatment of *H. contortus*, achieving a FECR of more than 95% and albendazole drugs yielded a FECR of less than 95% (Lin et al., 2016).

### *In Vitro* Test

Up to now, three studies have used EHA to test anthelmintic resistance in trichostrongyloid nematodes including *H. contortus* in China. Wang et al. (2000) used EHA to detect albendazole resistance in *H. contortus* on a sheep farm in Jiangsu province. The results showed that ED50 was 0.0602 μg/mL, which were about three times as the sensitive strain, but it was less than 0.1 μg/mL, suggested by World Association for the Advancement of Veterinary Parasitology (WAAVP), indicating no resistance was present. Zhao et al. (2010) detected benzimidazole resistance in trichostrongyloid nematodes in Urumqi region through EHA method. Resistance was present in two of four goat farms with ED50 of 0.270 and 0.293 μg/mL, respectively. *H. contortus* and *Teladorsagia* spp. were the dominant nematodes. Xia and Wang (2010) utilized the modified EHA to detect levamisole resistance in *H. contortus* in a goat farm in Shanghai, China. The ED50 was 0.295 and 0.406 μg/mL after egg hatch for 6 and 9 h, respectively, consistent with the ED50 of susceptible *H. contortus* described in a previous report (Dobson et al., 1986), indicating that no levamisole resistance was present in this farm.

### Molecular Test to Detect BZ Resistance

Three different SNPs in the isotype-1 β-tubulin gene known as F167Y (TTC to TAC) (Silvestre and Cabaret, 2002), E198A (GAA to GCA) (Ghisi et al., 2007; Rufener et al., 2009) and F200Y (TTC to TAC) (Kwa et al., 1994, 1995) have been demonstrated to be associated with BZ resistance in *H. contortus*. The alteration in nucleotide sequence has led to the change of protein structure and reduced the affinity of BZ molecules to β-tubulin (Prichard, 2001). Based on the detection of SNPs associated with BZ resistance, four studies were conducted in *H. contortus* in China.

A multiplex PCR was developed to detect the SNP F200Y in the isotype-1 β-tubulin gene associated with BZ resistance in five *H. contortus* populations including two populations from sheep (Shihezi and Yining of Xinjiang province) and three populations from goats (Wuhe of Anhui province, Nanjing and Xuzhou of Jiangsu province), revealing that resistant genotype was not present in any studied *H. contortus* populations (Bo and Li, 2005). Meanwhile, another study employing PCR-SSCP to detect the F200Y in field *H. contortus* population of sheep in Inner Mongolia discovered that homozygous susceptible genotype was in the majority (Hao, 2007). Furthermore, one study utilized PCR-RFLP method for detection of F200Y in *H. contortus* populations of sheep in Ningxia and Inner Mongolia province suggesting the similar case that homozygous susceptible genotype was most frequent (Cai and Bai, 2009).

In China, BZs has been broadly used to control worm load, resistance has been emerged in different regions of China, which has been reported recently (Zhang et al., 2016) by detecting all three known SNPs in the isotype-1 β-tubulin gene (F167Y, E198A, and F200Y) in *H. contortus* from eight populations in China including four populations from sheep (Hebei, Heilongjiang, Inner Mongolia, and Liaoning) and another four populations from goats (Guangxi, Hubei, Shaanxi, and Yunnan) using PCR-coupled sequencing. Five out of six genotypes were identified from 192 *H. contortus* adult males with SNP E198A (GCA) and/or F200Y (TAC). Sequence analysis revealed resistant allele frequencies were 0–70% and 0–31% for E198A and F200Y, respectively; however, F167Y was not detected in any populations. Genetic analysis showed that F200Y had multiple origins and E198A had two distinct origins in Chinese *H. contortus* populations. They concluded that BZ resistance is prevalent in different regions of China (Guangxi, Inner Mongolia, Liaoning, and Yunnan), therefore proper monitoring and control strategy of BZ resistance should be focused.

It has been proven that molecular methods could be used as a diagnostic tool to detect BZ resistance present in a population, even if it is not be able to evaluate the resistance at the quantitative level comparable to FECRT (Kotze and Prichard, 2016). Actually, the molecular tests and the biological tests should be used in combination in the field. Until now, there is no clear evaluation of the application of the molecular tests to detect BZ resistance in field samples containing lots of species of nematodes. Beyond that, the molecular tests have not been utilized in fecal samples containing nematode eggs directly, which could save a lot of time (Roeber et al., 2013b). From now on, whether using existing methods or developing new molecular methods, the two issues should be studied in depth.

### Detection of SNPs in IVM Resistant *H. contortus*

IVM also belongs to the major classes of anthelmintics and has excessively been used, which led to widespread resistance in nematodes, particularly in *H. contortus*. Other than several genes which code for IVM target and efflux pumps, IVM resistance is believed to be highly multi-genic in nature (Lespine et al., 2012; Redman et al., 2012). In order to discover more SNPs associated with IVM resistance in *H. contortus*, Luo et al. (2017) looked for SNPs across the whole genome in both susceptible and resistant isolates of *H. contortus* maintained in goats by using 2b-RAD sequencing method. 2962 SNPs were found in susceptible isolates and 2667 SNPs in resistant isolates. Similar and comparatively lower genetic variations were seen within either resistant or susceptible strains. However, comparison between the two strains revealed 208 SNPs with significant difference, out of which 24 SNPs were in CDS region of the nine genes. These were likely to direct IVM selection and seven out of these nine candidate genes were predicted to code for some proteins which could play vital role in IVM target or efflux pump proteins and for transcriptional regulation proteins as well as for component proteins of receptor complexes such as membrane or neuromuscular cells. These genes were believed to be involved in IVM resistance. The findings from this study suggested that genes which are involved in directing IVM selection and associated with IVM resistance in *H. contortus* can be identified by using genome-wide SNP analysis employing 2b-RAD sequencing technique.

## Studies on Diapause-Related Genes of *Haemonchus contortus* in China

*Haemonchus contortus* can enter into the arrested development (diapause) during the infective L3s (iL3s) of free-living life stage or early L4 of the parasite stage to protect them against hostile environment. Understanding this developmental change might identify key molecules as new drug targets and provide new insight into prevention and control of *H. contortus.* Here, we reviewed the studies on 10 genes which are related to diapause of *H. contortus* and their structures and functions have been characterized at the molecular level (**Table 1**).

**Table 1:** Information on studied diapause related genes of *Haemonchus contortus*.

Gene	Length (aa)	Promoter expression pattern		Highest transcription	Functions	Reference
		*H. contortus*	*C. elegans*			
*Hc-daf-2*	1404	AWA	AWA, ADF, ASH, nerve ring	iL3	Insulin receptor; partially rescue *Ce-daf-2* mutant; related in the transition to parasitism	Li et al., 2014b
*Hc-age-1*	1156	Intestine	Amphidial neurons, intestine	Eggs, iL3, FA^a^	A subunit of PI3Ks; interaction with *Hc-aap-1*	Li et al., 2014b
*Hc-aap-1*	427	Anterior intestine	Amphidial and phasmidial neurons	Eggs, FA, iL3, FL4s^b^	A subunit of PI3Ks; interaction with *Hc-age-1*	Li et al., 2014b
*Hc-pdk-1*	576	Head and tail neurons, intestine	Head neurons, pharynx, intestine, hypodermal cells	iL3	Similar to *Ce-pdk-1*, promote arrested development	Li F.C. et al., 2016
*Hc-ubq*	128				Resistance desiccation through the ubiquitin-protease system	Yang et al., 2015
*Hc-gst*	205				Resistance desiccation through the enzymatic antioxidant pathway	Yang et al., 2015
*Hc38*	592				Affect larvae development	He et al., 2011
*Hc-fau-a/b*	130/107	Intestine	Intestine, pharynx, muscle, neurons	L4s (lowest)	Lifespan; body size; reproduction	Yan B. et al., 2014
*Hc-maoc-1*	299	intestine (IFA)	Intestine	L4	Peroxisomal β-oxidation; lifespan; body length	Ding et al., 2017
*Hc-daf-22*	533	Pharynx, excretory cell, intestine	Pharynx, intestine, hypodermis	L3, L4	Peroxisomal β-oxidation and the development in *H. contortus*	Guo et al., 2016
*Hc-hsp70*	646	Intestine	Multiple tissues^c^	eggs	Homologous to *C. elegans hsp-1*, related to invasion and survival of nematodes	Zhang et al., 2013

*^a^FA: female adult; ^b^FL4s: female L4s; ^c^Multiple tissues: pharynx, anal depressor muscle, body wall muscle, excretory cell and in unidentified cells besides the intestine.*

It was found that infective L3 (iL3s) of parasitic nematodes have significant similarities in anatomy, behavior and biology with the dauer stage of free-living nematode *Caenorhabditis elegans*. Thus, the “dauer hypothesis” deems that the iL3 of parasitic nematodes is developmentally and functionally analogous to the dauer stage of *C. elegans* and is regulated by similar molecular mechanism (Hotez et al., 1993; Blaxter, 1998; Bürglin et al., 1998; Hu, 2007). Insulin-like signaling pathway governs dauer development in *C. elegans* and is conserved in several species of parasitic nematodes including *H. contortus*. Five genes were found to belong to the insulin-like signaling pathway in *H*. *contortus*: fork head transcription factor encoding gene *Hc-daf-16* (Hu et al., 2010), insulin receptor encoding gene *Hc-daf-2* (Li et al., 2014b), phosphoinositide 3-kinases (PI3Ks) component catalytic subunit encoding gene *Hc-age-1* and regulatory subunit encoding gene *Hc-aap-1* (Li et al., 2014a) and phosphoinositide dependent protein kinase-1 gene *Hc-pdk-1* (Li F.C. et al., 2016). These genes all had expression patterns in the intestine that were consistent with those of the homologous genes of *C. elegans* correspondingly, indicating a similar location where they regulate the growth and development. It was found that *Hc-daf-2* can partially rescue the *C. elegans daf-2* mutant (CB1379) worm (Li et al., 2014b), suggesting to some extent, *Hc-daf-2* has functional similarity to *Ce-daf-2*. Downstream of *Hc-*DAF-2 is the PI3K kinase with two subunits encoded by *Hc-age-1* and *Hc-aap-1*, respectively. These two molecules can interact strongly with each other demonstrated by yeast two-hybrid method. However, *Hc-age-1* can’t rescue *age-1-*deficient strain of *C. elegans* (CY246) like *Ce-age-1*. The possible interpretation may be because that the exogenous *Hc-age-1* subunit can’t bind to the endogenous *Ce-aap-1* subunit *in vivo* to constitute the functional PI3K (Li et al., 2014a). Interestingly, *Hc-pdk-1* displayed conserved functional domains which are crucial for the phosphorylation of downstream signaling. Its function may be similar to *Ce-pdk-1*, promoting arrested development (Li F.C. et al., 2016). At the transcriptional level, RNAseq analysis showed that these four genes have highest transcriptional expression levels in iL3, which indicated that these genes could play an important role in regulating iL3 against external unfavorable environment. In summary, the findings from these studies provide evidences of the functional conservation of insulin-like signaling between *H. contortus* and *C. elegans*. Additionally, the reconstructed insulin-like signaling pathway of *H. contortus* from transcriptomic and genomic data sets for this nematode by a bioinformatic approach further confirmed its existence in *H. contortus* (Mohandas et al., 2016a). However, although the basic skeleton of insulin-like signaling pathway in the *H. contortus* is built, the mechanism about how these genes and this pathway function in the development of *H. contortus* need to be further studied in the future.

In China, in addition to the discovery of insulin signaling pathways, researchers are currently working on other signaling pathways such as TGF-beta signaling pathway which is also vital for normal development of *C. elegans* and it is important to understand their roles in *H. contortus* diapause in the iL3. What’s more, as *H. contortus* iL3s are able to protect themselves from desiccation, genes involved in this biological process were also identified and their functions were studied. The mRNA differential display RT-PCR was used to screen differentially expressed genes in L3s upon desiccation, among the 58 differentially expressed gene transcripts, two transcripts with highest transcription were named *Hc-ubq* and *Hc-gst* based on their homologs ubiquitin in *C. elegans* and glutathione-S-transferase in *H. contortus*, respectively. Silencing *Hc-ubq* or *Hc-gst* by RNAi in L3s of *H. contortus* reduced the survival rate, suggesting that they may contribute to the nematode desiccation tolerance (Yang et al., 2015). In addition, silencing *Hc38*, which was first discovered by Northern blot and highly expressed in the intestinal microvilli by *in situ* localization (Hartman et al., 2001), by soaking iL3 of *H. contortus* in dsRNA which were then used to infect sheep reduced the amount of egg and worm burden by 50 and 48.6%, respectively, suggesting *Hc38* is involved in the development of *H. contortus* (He et al., 2013).

In addition to diapause in iL3, *H. contortus* can enter into diapause in early L4 in the parasitic stage. The larvae with arrested development have shorter-length, reduced body metabolic rate and rod-shaped crystals appeared in the intestinal tract. Staying as early L4 in the host and not spawning can protect them against the cold weather. For diapause in this stage, three genes were found highly expressed. They are *Hc-daf-22* (encoding 3-ketoacyl-CoA thiolase), *Hc-maoc-1* (encoding enoyl-CoA hydratase) and *Hc-hsp-70* (encoding heat shock protein 70). *Hc-daf-22* and *Hc-maoc-1* shared similar characteristics and functions with their homologs in *C. elegans, Ce-daf-22* and *Ce-maoc-1*, respectively, and may play important roles in peroxisomal β-oxidation and the development of *H. contortus* (Guo et al., 2016; Ding et al., 2017). *Hc-hsp-70* is highly conserved in other nematodes, especially in *Caenorhabditis*. Over-expression of *Hc-hsp-70* induced down-regulation of *hsp-1* of *C. elegans*, which suggested that *Hc-hsp-70* might have similar function to *Ce-hsp-1* of involvement in the parasite invasion and survival (Zhang et al., 2013). *Hc-fau*, a homolog of human *fau* and *C. elegans Ce-rps30* (encoding ribosomal protein S30), have a conserved ribosome protein S30 domain and a diverged ubiquitin-like (UBiL) protein domain. The S30 is mainly expressed in the nucleus while the UBiL is strongly expressed in the cytoplasm. Both of them have effect on egg-laying and life span of *C. elegans*, suggesting that they may have potential functions in regulating L4 diapause in *H. contortus* (Yan B. et al., 2014).

## Vaccine Development of *Haemonchus contortus*

Excessive use of anthelmintics for prevention and control of parasitic nematode diseases threatening human and animals have caused serious issues regarding anthelmintic resistance and drug residues worldwide (Saddiqi et al., 2011; Papadopoulos et al., 2012). As an alternative strategy, vaccine is a possible option for controlling parasitic nematodes including *H. contortus* (Bassetto and Amarante, 2015). Substantial progresses have been made during the past two decades in identifying several potential antigens from *H. contortus* as they can stimulate prominent levels of protective immunity in the immunized hosts (Knox et al., 2003; Tak et al., 2015). Here, we summarized the molecular properties and the protective efficacy of principal candidate antigens by using recombinant subunit vaccine and DNA vaccination. We hope that these insights will contribute to the study of molecular explorations of antigens and promote the development of *H. contortus* vaccines research in the future.

### Recombinant Subunit Vaccine

Gene recombination, a significant biological technology has particularly attracted close attention in the development of commercial vaccine. Currently, partial protection has been conferred by immunization with recombinant antigens of *H. contortus*. The best characterized candidate antigen of *H. contortus*, termed H11, was a 110 kDa integral membrane glycoprotein complex (Smith et al., 1993, 1997). It was a highly effective immunogen [>90% reduction in fecal egg counts (FECs), >75% reduction in worm burden] against *H. contortus* challenge (Newton and Munn, 1999). Therefore, some scientists are expecting to develop recombinant vaccine for future large-scale production facilitating the practical use to achieve immune protection of the hosts. The H11-1 and H11-2 of H11 forms had been expressed in *Escherichia coli*, which showed aminopeptidase activity with the enzyme activity of H11-2 significantly higher than that of H11-1 (Yan and Li, 2006). Subsequently, the 4- to 6-months-old goats were immunized twice with phosphate-buffered saline (PBS), recombinant H11-1, H11-2 and mixture of H11-1 and H11-2. Immunization with mixture of H11-1 and H11-2 conferred partial protection (a 29% reduction in FECs and a 18% reduction in worm burden) compared with other groups (Yan et al., 2007). In addition, the three fragments (H11-1, -2 and -3) of H11 gene were inserted into yeast expression vector and recombinant plasmids were transformed into *Pichia pastoris* X-33 by lithium chloride method. Transcriptions were detected by RT-PCR and the glycosylated proteins were demonstrated by SDS–PAGE and Western blot (Yan and Li, 2005). However, no protection experiment was carried out for the recombinant molecule containing three H11 isoforms.

Except for *E. coli* and *P. pastoris, C. elegans* was also attempted as a vehicle to express H11 to improve the protection. In such experiment, a 1517 bp 5′ flanking region and part of the first exon of the H11 gene of *H. contortus* and homologous gene of *C. elegans* were, respectively, sub-cloned into the upstream region of green fluorescence protein reporter gene in the pPD95.77 vector. The recombinant plasmids were microinjected into the gonads of *C. elegans*, respectively. The results demonstrated different transcriptional expression patterns driven by their promoter regions from free-living and blood-sucking nematode species, respectively, highlighting the availability of *C. elegans* as a heterologous system to study the biological characteristics of H11 isoforms (Zhou et al., 2010). Using this expression system, Zhou et al. (2014) then produced recombinant Trans-HPS (a 1710 bp fragment of isoform H11 gene). Immunization with the crude Trans-HPS extracted from transgenic worms resulted in 38% reduction in FECs and 25% reduction in worm burden. However, *E. coli* expressed, a gene fragment from nt 670 bp to 1710 bp of isoform H11 gene failed to protect sheep in immunization experiments (Zhou et al., 2014).

Apart from the potential antigen H11, recombinant Hco-gal-m/f (derived from male and female worms, respectively) of galectin from *H. contortus*, expressed in *E. coli* and co-administered with Freund’s adjuvant, vaccination with 200 μg protein reduced fecal egg output and worm burdens by 48 and 46%, respectively. The findings suggested that vaccination with a combination of recombinant Hco-gal-m/f proteins had a role in protecting goats against *H. contortus* infection (Sun et al., 2007b).

### DNA Vaccination

DNA vaccination is a technique for protecting against diseases by injection with genetically engineered DNA so cells directly express antigens and animals immunized with DNA vaccine can produce protective immune responses. DNA vaccines have potential advantages over conventional vaccines, including the ability to induce a wider range of different immune responses. It represents a novel approach for the control of infectious parasitic diseases. For example, direct injection of a naked plasmid DNA vaccine encoding an exogenous antigen led to the plasmid uptake and antigen expression, resulted in the induction of antigen-specific immune responses (Tang et al., 1992; Egan and Israel, 2002). For *H. contortus*, several reports have been published and described partial protection in goats following DNA vaccination (**Table 2**). Immunization of 8- to 10-month-old goats with DNA vaccine encoding HC29, an *H. contortus* glutathione peroxidase (GPX), induced specific antibodies and partial immune protection (a 36% reductions in FECs and worm burdens) compared with goats which had received only PBS (Sun et al., 2011). Vaccination of goats with DNA vaccines containing three fragments encoding sections of H11-1 and caprine interleukin-2 (IL-2), resulted in high levels of specific serum immunoglobulin G (IgG), IgA, CD4^+^ T lymphocytes and CD8^+^ T lymphocytes as well as reductions of fecal egg output and abomasal worm burdens of 57 and 47%, respectively (Zhao et al., 2012).

**Table 2:** Summary of the vaccine protection trials for *Haemonchus contortus* by DNA vaccines.

Antigen	Amount of antigen	Number of vaccinations given	Effect on FEC^a^	Effect on worm burden^b^	Challenge	Reference
HC29	500 μg	2	36%	36%	5000 L3	Sun et al., 2011
H11-1+IL-2	300 μg	2	57%	47%	5000 L3	Zhao et al., 2012
HcGAPDH	500 μg	2	35%	38%	5000 L3	Han et al., 2012
Dim-1	500 μg	2	35%	35%	5000 L3	Yan et al., 2013
Actin	100 μg	2	34%	33%	5000 L3	Yan R. et al., 2014

*In all trials, goats (8–10 months of age) received intramuscular injection of the vaccines, and every injection volume was equally divided between two injection sites in the thigh and shoulder muscles.*

*^a^Reduction in fecal egg count (FEC) compared with PBS control group.*

*^b^Reduction in worm burden compared with PBS control group.*

In a further test, proteins of critical functions in worm physiology have been registered as the goal of DNA vaccines. For instance, glyceraldehyde-3-phosphate dehydrogenase in *H. contortus* (HcGAPDH) as the DNA vaccine had been tested for protection against experimental *H. contortus* infections in goats. The result showed that DNA vaccine induced significant peripheral and local mucosal immune responses, promoted the proliferations of CD4^+^ T and B lymphocytes, but only provided partial protection (a 35% reduction in FECs and a 38% reduction in worm burden) compared with control groups (Han et al., 2012). Vaccination with disorganized muscle family member (Dim-1) DNA vaccine could offer slightly better protection (a 46% reduction in FECs and a 51% reduction in worm burden) to corresponding infection in goats (Yan et al., 2013). Studies had also suggested that *H. contortus* actin DNA vaccine could induce partial immune response (approximately 34% reduction in FECs and worm burden), but no difference was found in CD4^+^ T lymphocytes, B lymphocytes and eosinophils between actin group and PBS control group after challenge (Yan R. et al., 2014).

## Immuno-Regulation of Goat PBMC

To discover useful vaccine antigens, it is crucial to understand the mechanisms of the immune regulation. Some studies have been carried out in China to explore the mechanism of immuno-regulation during parasite invasion by investigating the interaction between parasite molecules and host cells. To do so, the host peripheral blood mononuclear cells (PBMC) from goat blood were isolated by venipuncture. PBMC is actually a mixture of subpopulations of functional cells, which mainly includes lymphocytes (T cells, B cells, and NK cells), monocytes, and dendritic cells (Wang et al., 2014a). All of the subpopulations are critical to reflect interaction mechanism between the host cells and many important parasite molecules. For example, *H. contortus* galectin peptides recombinant Hco-gal-m/f (rHco-gal-m/f) were cultivated with PBMC of goats, investigated the effect of rHco-gal-m/f to induce apoptosis in the PBMC (Sun et al., 2007a). A combined proteomic and transcriptomic analysis had also been performed to understand the mechanisms underlying the immunomodulation induced by rHco-gal-m/f of *H. contortus* on goat PBMC. The findings demonstrated that rHco-gal-m/f could bind to the surface of goat PBMC and behaved as the suppressors of inflammatory response to facilitate the immune evasion of *H. contortus* (Wang et al., 2014b).

On the basis of the above study, further work by yeast two-hybrid screening found two Hco-gal-m and -f binding partners, transmembrane protein 147 (TMEM147) and transmembrane protein 63A (TMEM63A). The interaction of galectin with TMEM147 mainly mediated cell proliferation, cell apoptosis, and cytokine transcription in goat PBMC. This membrane protein, together with TMEM63A, was also involved in the regulation of galectin on phagocytosis and nitric oxide production of goat PBMC. However, TMEM63A might play a greater role than TMEM147 in the regulation of galectin in the migration and IFN-γ transcription of goat PBMC. These findings provided new perspective to the elucidation of the mechanisms involved in immune evasion by nematodes and in parasite–host interactions (Yuan et al., 2015; Li Y. et al., 2016).

Excretory and secretory products as vaccine candidate antigens of *H. contortus* contain various proteins, which can stimulate or depress the host immune response and are involved in the pathogenesis of the worms. Research showed that the *H. contortus* excretory and secretory products (HcESPs) displayed suppressive potential on the goat PBMC *in vitro*. HcESPs inhibited the productions of IL-4, IFN-γ, increased the suppressive cytokine IL-10, enhanced the inflammatory modulator IL-17, suppressed the production of chemical factor nitric oxide, decreased the cell proliferation and activated the cell migration (Gadahi et al., 2016d). Meanwhile, Gadahi et al. (2016c) also reported the interaction of proteins from HcESPs at different developmental stages to goat PBMC *in vivo* using liquid chromatography-tandem mass spectrometry. A total of 407 HcESPs that interacted with goat PBMC at different time points were identified. This study identified the secreted *H. contortus* 14-3-3 protein as a goat PBMC-interacting protein in all parasitic stages. In a follow-up study, recombinant protein of *H. contortus* 14-3-3 isoform 2 (rHcftt-2) decreased the production of IL-4 and suppressed the proliferation of goat PBMC *in vitro* (Gadahi et al., 2016a). In addition, a 24 kDa *H. contortus* excretory/secretory protein (HcES-24) also showed to have important antigenic function. The immune interactions between recombinant protein of HcES-24 and goat PBMC demonstrated that IL-4, IL-10, IL-17 and cell migration were increased. Nevertheless, the interaction significantly suppressed the PBMC proliferation and NO production. The findings showed that the rHcES-24 played important regulatory effects on the goat PBMC (Gadahi et al., 2016b).

## Conclusion

*Haemonchus contortus* has the capacity to cause huge economic losses in China. Over the past two decades, studies in China on *H. contortus* were carried out covering a range of research areas including epidemiological survey, population genetic studies, detection of anthelmintic resistance, diapause genes related with development, vaccine development and immuno-regulation. Until 2017, most provinces in China have reported *H*. *contortus* infection with high prevalence in goats, sheep, and cattle. It was found that high genetic variation existed in Chinese *H. contortus* and most genetic variations were distributed within population, and there was high gene flow among populations, indicating drug resistance could be rapidly spread. Resistance against BZ was detected in several *H. contortus* populations, emphasizing the importance of detection and monitoring drug resistance for optimizing control strategy. Encouraging is the emergence of studies on screening the SNPs in the genome of *H. contortus* resistant against ivermectin as ivermectin resistance has been found in many places in China discovered by a recent nation-wide research project (funded by Special Fund for Agro-scientific Research in the Public Interest). Fundamental studies into structure and function of diapause related genes and the mechanism of immuno-regulation will provide insights into the molecular mechanism on developmental change and immune invasion of the parasite. In addition, vaccine development has also been attempted. These achievements are visible and provide valuable bases for future research exploration in these areas.

In addition to the above mentioned research areas, more research work should be carried out to assess the relationship between the infection rate/intensity and the severity of haemonchosis in order to implement more cost-effective control strategy. The usefulness of molecular methods in detecting drug resistance in the field should be evaluated in order to detect the emergency of drug resistance as early as possible and monitor its development to decrease the adverse influence of drug resistance. The extent of drug resistance should be investigated in a nationwide scale to understand the seriousness of drug resistance in China for which is still almost unknown. More sensitive and specific methods should be developed for use in the field and for detecting mixed infections. Finally, more fundamental studies relating to the biology of this parasite and its interaction with hosts of domestic animals are still needed to better understand the biology of *H. contortus* and the disease it causes in order to establish more useful diagnostic methods, develop effective drugs and vaccines, which will contribute to the preventing and controlling *H. contortus* in China.

## Author Contributions

MH conceived and designed the project. CW, FL, ZZ, XY, AA, and MH contributed to the writing of the manuscript with the input from XL and AD. All authors read and approved the final manuscript.

## Conflict of Interest Statement

The authors declare that the research was conducted in the absence of any commercial or financial relationships that could be construed as a potential conflict of interest.

## Footnotes

1 http://data.stats.gov.cn/index

## References

[B1] Bassetto C. C. Amarante A. F. (2015). Vaccination of sheep and cattle against haemonchosis. *J. Helminthol.* 89 517–525. 10.1017/s0022149x15000279 25891536

[B2] Blaxter M. (1998). *Caenorhabditis elegans* is a nematode. *Science* 282 2041–2046. 10.1126/science.282.5396.2041 9851921

[B3] Blitz N. M. Gibbs H. C. (1971). Morphological characterization of the stage of arrested development of *Haemonchus contortus* in sheep. *Can. J. Zool.* 47 991–995. 10.1139/z71-151 5118671

[B4] Bo X. W. Li X. R. (2005). Multiplex PCR detection of allele on benzimidazole-resistance or -susceptibility in natural populations of *Haemonchus contortus*. *Sci. Agric. Sin.* 38 826–830.

[B5] Britton C. Roberts B. Marks N. D. (2016). Functional genomics tools for *Haemonchus contortus* and lessons from other helminths. *Adv. Parasitol.* 93 599–623. 10.1016/bs.apar.2016.02.017 27238014

[B6] Bürglin T. R. Lobos E. Blaxter M. L. (1998). *Caenorhabditis elegans* as a model for parasitic nematodes. *Int. J. Parasitol.* 28 395–411. 10.1016/S0020-7519(97)00208-7 9559358

[B7] Cai K. Z. Bai J. L. (2009). Analysis of alleles resistant to benzimidazoles in *Haemonchus contortus* in sheep by PCR-RFLP. *Chin. Vet. Sci.* 39 685–689.

[B8] Cai K. Z. Yang X. Y. Wang X. L. Hao C. Yang A. Zhao Y. F. (2007). Investigation on resistance of gastrointestinal nematodes in sheep and goats to anthelmintics in Ningxia, China. *Vet. Sci. China* 2 1120–1123.

[B9] Cao X. M. Liu Y. Fang R. M. Hu M. Q. (1995). Investigation and deworming assays of gastrointestinal nematode infection in goats in Jianli County. *J. Hunan Agric. Coll.* 21 75–78.

[B10] Chen H. M. Wen G. B. Bian R. L. Qian C. L. Ye B. Bai J. M. (2000). Investigation of parasite in goats. *China Herbivores* 2 34–35.

[B11] Ding H. Shi H. Shi Y. Guo X. Zheng X. Chen X. (2017). Characterization and function analysis of a novel gene, *Hc-maoc-1*, in the parasitic nematode *Haemonochus contortus*. *Parasit. Vectors* 10:67 10.1186/s13071-017-1991-1 PMC5294872 28166831

[B12] Dobson R. J. Donald A. D. Waller P. J. Snowdon K. L. (1986). An egg-hatch assay for resistance to levamisole in trichostrongyloid nematode parasites. *Vet. Parasitol.* 19 77–84. 10.1016/0304-4017(86)90034-8 3962165

[B13] Egan M. A. Israel Z. R. (2002). The use of cytokines and chemokines as genetic adjuvants for plasmid DNA vaccines. *Clin. Appl. Immunol. Rev.* 2 255–287. 10.1016/S1529-1049(02)00051-X

[B14] Emery D. L. Hunt P. W. Le J. L. (2016). *Haemonchus contortus*: the then and now, and where to from here? *Int. J. Parasitol.* 46 755–769. 10.1016/j.ijpara.2016.07.001 27620133

[B15] Feng X. Y. Zhai T. Fu M. Z. (2016). Diagnose, infection investigation and drug resistance detection of *Haemonchus contortus* in a dairy goat farm. *Acta Ecol. Anim. Domast.* 37 61–64.

[B16] Gadahi J. A. Ehsan M. Wang S. Zhang Z. Wang Y. Yan R. (2016a). Recombinant protein of *Haemonchus contortus* 14-3-3 isoform 2 (rHcftt-2) decreased the production of IL-4 and suppressed the proliferation of goat PBMCs *in vitro*. *Exp. Parasitol.* 171 57–66. 10.1016/j.exppara.2016.10.014 27751769

[B17] Gadahi J. A. Li B. Ehsan M. Wang S. Zhang Z. Wang Y. (2016b). Recombinant *Haemonchus contortus* 24 kDa excretory/secretory protein (rHcES-24) modulate the immune functions of goat PBMCs *in vitro*. *Oncotarget* 7 83926–83937. 10.18632/oncotarget.13487 27893414 PMC5356635

[B18] Gadahi J. A. Wang S. Bo G. Ehsan M. Yan R. Song X. (2016c). Proteomic analysis of the excretory and secretory proteins of *Haemonchus contortus* (HcESP) binding to goat PBMCs *in vivo* revealed stage-specific binding profiles. *PLoS ONE* 11:e0159796 10.1371/journal.pone.0159796 PMC4965049 27467391

[B19] Gadahi J. A. Yongqian B. Ehsan M. Zhang Z. C. Wang S. Yan R. F. (2016d). *Haemonchus contortus* excretory and secretory proteins (HcESPs) suppress functions of goat PBMCs *in vitro*. *Oncotarget* 7 35670–35679. 10.18632/oncotarget.9589 27229536 PMC5094953

[B20] Ghisi M. Kaminsky R. Maser P. (2007). Phenotyping and genotyping of *Haemonchus contortus* isolates reveals a new putative candidate mutation for benzimidazole resistance in nematodes. *Vet. Parasitol.* 144 313–320. 10.1016/j.vetpar.2006.10.003 17101226

[B21] Guo X. Zhang H. Zheng X. Zhou Q. Yang Y. Chen X. (2016). Structural and functional characterization of a novel gene, *Hc-daf-22*, from the strongylid nematode *Haemonchus contortus*. *Parasit. Vectors* 9:422 10.1186/s13071-016-1704-1 PMC4966567 27472920

[B22] Han K. Xu L. Yan R. Song X. Li X. (2012). Vaccination of goats with glyceraldehyde-3-phosphate dehydrogenase DNA vaccine induced partial protection against *Haemonchus contortus*. *Vet. Immunol. Immunopathol.* 149 177–185. 10.1016/j.vetimm.2012.06.016 22771197

[B23] Han X. Y. Qiong R. Canjue Z. M. (1984). Investigation of sheep parasite in Bayi area of Tibet. *Jilin Anim. Husb. Vet. Med.* 4 25–31.

[B24] Hao C. (2007). *Study of Detection of Benzimidazoles Resistance and PCR-SSCP Analysis in Gastrointestinal Nematodes of Small Domestic Ruminants.* Master’s thesis, Inner Mongolia Agricultural University Hohhot.

[B25] Hartman D. Donald D. R. Nikolaou S. Savin K. W. Hasse D. Presidente P. J. (2001). Analysis of developmentally regulated genes of the parasite *Haemonchus contortus*. *Int. J. Parasitol.* 31 1236–1245. 10.1016/S0020-7519(01)00248-X 11513893

[B26] He Y. H. Wang X. H. Bo X. W. Chen N. Y. Zhang P. Zhang X. Y. (2013). Effect of Hc38 gene silencing on the development of L3 larvae of *Haemonchus contortus*. *Acta Agric. Boreali Occident. Sin.* 22 19–26.

[B27] He Y. H. Wang X. H. Bo X. W. Chen N. Y. Zhang X. Y. Kang L. C. (2011). Expression purification and antigenic identification of recombinant Hc38 protein of *Haemonchus contortus*. *Xinjiang Agric. Sci.* 48 750–754.

[B28] Hoberg E. P. Zarlenga D. S. (2016). Evolution and biogeography of *Haemonchus contortus*: linking faunal dynamics in space and time. *Adv. Parasitol.* 93 1–30. 10.1016/bs.apar.2016.02.021 27238001

[B29] Hotez P. Hawdon J. Schad G. A. (1993). Hookworm larval infectivity, arrest and amphiparatenesis: the *Caenorhabditis elegans* Daf-c paradigm. *Parasitol. Today* 9 23–26. 10.1016/0169-4758(93)90159-D 15463660

[B30] Hu M. Lok J. B. Ranjit N. Massey H. C. Jr. Sternberg P. W. Gasser R. B. (2010). Structural and functional characterisation of the fork head transcription factor-encoding gene, *Hc-daf-16*, from the parasitic nematode *Haemonchus contortus* (Strongylida). *Int. J. Parasitol.* 40 405–415. 10.1016/j.ijpara.2009.09.005 19796644 PMC2853935

[B31] Hu P. J. (2007). “Dauer,” in *WormBook* ed. The C. elegans Research Community (Pasadena, CA: WormBook), 1–19. 10.1895/wormbook.1.144.1

[B32] Jiang B. Wu S. H. Lin L. Li C. S. Zhang S. Z. (2017). An epidemiological survey of the major helminths of goats in Fujian province. *Fujian J. Anim. Husb. Vet.* 39 1–4.

[B33] Kaplan R. M. (2004). Drug resistance in nematodes of veterinary importance: a status report. *Trends Parasitol.* 20 477–481. 10.1016/j.pt.2004.08.001 15363441

[B34] Kaplan R. M. Vidyashankar A. N. (2012). An inconvenient truth: global worming and anthelmintic resistance. *Vet. Parasitol.* 186 70–78. 10.1016/j.vetpar.2011.11.048 22154968

[B35] Knox D. P. Redmond D. L. Newlands G. F. Skuce P. J. Pettit D. Smith W. D. (2003). The nature and prospects for gut membrane proteins as vaccine candidates for *Haemonchus contortus* and other ruminant trichostrongyloids. *Int. J. Parasitol.* 33 1129–1137. 10.1016/S0020-7519(03)00167-X 13678629

[B36] Kotze A. C. Hunt P. W. Skuce P. von Samson-Himmelstjerna G. Martin R. J. Sager H. (2014). Recent advances in candidate-gene and whole-genome approaches to the discovery of anthelmintic resistance markers and the description of drug/receptor interactions. *Int. J. Parasitol. Drugs Drug Resist.* 4 164–184. 10.1016/j.ijpddr.2014.07.007 25516826 PMC4266812

[B37] Kotze A. C. Prichard R. K. (2016). Anthelmintic resistance in *Haemonchus contortus*: history, mechanisms and diagnosis. *Adv. Parasitol.* 93 397–428. 10.1016/bs.apar.2016.02.012 27238009

[B38] Kwa M. S. Veenstra J. G. Roos M. H. (1994). Benzimidazole resistance in *Haemonchus contortus* is correlated with a conserved mutation at amino acid 200 in beta-tubulin isotype 1. *Mol. Biochem. Parasitol.* 63 299–303. 10.1016/0166-6851(94)90066-3 7911975

[B39] Kwa M. S. Veenstra J. G. Van Dijk M. Roos M. H. (1995). Beta-tubulin genes from the parasitic nematode *Haemonchus contortus* modulate drug resistance in *Caenorhabditis elegans*. *J. Mol. Biol.* 246 500–510. 10.1006/jmbi.1994.0102 7877171

[B40] Lespine A. Menez C. Bourguinat C. Prichard R. K. (2012). P-glycoproteins and other multidrug resistance transporters in the pharmacology of anthelmintics: prospects for reversing transport-dependent anthelmintic resistance. *Int. J. Parasitol. Drugs Drug Resist.* 2 58–75. 10.1016/j.ijpddr.2011.10.001 24533264 PMC3862436

[B41] Li F. C. Gasser R. B. Lok J. B. Korhonen P. K. He L. Di W. D. (2016). Molecular characterization of the *Haemonchus contortus* phosphoinositide-dependent protein kinase-1 gene (*Hc-pdk-1*). *Parasit. Vectors* 9:65 10.1186/s13071-016-1351-6 PMC4741024 26842781

[B42] Li F. C. Gasser R. B. Lok J. B. Korhonen P. K. Wang Y. F. Yin F. Y. (2014a). Exploring the role of two interacting phosphoinositide 3-kinases of *Haemonchus contortus*. *Parasit. Vectors* 7:498 10.1186/s13071-014-0498-2 PMC4233088 25388625

[B43] Li F. C. Lok J. B. Gasser R. B. Korhonen P. K. Sandeman M. R. Shi D. (2014b). *Hc-daf-2* encodes an insulin-like receptor kinase in the barber’s pole worm, *Haemonchus contortus*, and restores partial dauer regulation. *Int. J. Parasitol.* 44 485–496. 10.1016/j.ijpara.2014.03.005 24727120 PMC4516220

[B44] Li H. P. Deng L. J. Ren J. L. (2007). Treatment and diagnosis of haemonchosis in Guangzhong Milk Goat. *Anim. Husb. Vet. Med.* 39 43–44.

[B45] Li M. Jin A. Yang C. Y. Su J. Y. Chen D. Q. Nie K. (2001). Investigation of parasites of cattle and sheep in Jiangjin City. *Chin. J. Vet. Parasitol.* 9 29–33.

[B46] Li S. R. Ge C. R. Yang D. P. Wang M. Ye R. Q. Fu A. G. (2002). Investigation on parasites and parasitic diseases of cattle and sheep in Baishui River area of Zhanyi County. *Chin. J. Vet. Parasitol.* 10 30–33.

[B47] Li Y. Yuan C. Wang L. Lu M. Wang Y. Wen Y. (2016). Transmembrane protein 147 (TMEM147): another partner protein of *Haemonchus contortus* galectin on the goat peripheral blood mononuclear cells (PBMC). *Parasit. Vectors* 9:355 10.1186/s13071-016-1640-0 PMC4918192 27337943

[B48] Lin L. Jiang B. Wu S. H. Zhang S. Z. Lin S. Cai X. (2016). The insecticide effect comparison trial of different drugs to *Haemonchus contortus* in grazing goats. *Fujian J. Anim. Husb. Vet. Med.* 38 1–3.

[B49] Liu Y. Li F. Liu W. Dai R. S. Tan Y. M. He D. S. (2009). Prevalence of helminths in water buffaloes in Hunan Province, China. *Trop. Anim. Health Prod.* 41 543–546. 10.1007/s11250-008-9219-1 18704740

[B50] Luo J. Li J. W. Wu Y. H. (2005). Present situation and development countermeasures of goat industry in China. *Chin. J. Anim. Sci.* 41 55–57.

[B51] Luo X. Shi X. Yuan C. Ai M. Ge C. Hu M. (2017). Genome-wide SNP analysis using 2b-RAD sequencing identifies the candidate genes putatively associated with resistance to ivermectin in *Haemonchus contortus*. *Parasit. Vectors* 10:31 10.1186/s13071-016-1959-6 PMC5240194 28095895

[B52] Ma J. He S. W. Li H. Guo Q. C. Pan W. W. Wang X. J. (2014). First survey of helminths in adult goats in Hunan Province, China. *Trop. Biomed.* 31 261–269. 25134894

[B53] Mohandas N. Hu M. Stroehlein A. J. Young N. D. Sternberg P. W. Lok J. B. (2016a). Reconstruction of the insulin-like signalling pathway of *Haemonchus contortus*. *Parasit. Vectors* 9:64 10.1186/s13071-016-1341-8 PMC4741068 26842675

[B54] Mohandas N. Young N. D. Jabbar A. Korhonen P. K. Koehler A. V. Hall R. S. (2016b). The complement of family M1 aminopeptidases of *Haemonchus contortus*–biotechnological implications. *Biotechnol. Adv.* 34 65–76. 10.1016/j.biotechadv.2015.10.003 26597954

[B55] Newton S. E. Meeusen E. N. (2003). Progress and new technologies for developing vaccines against gastrointestinal nematode parasites of sheep. *Parasite Immunol.* 25 283–296. 10.1046/j.1365-3024.2003.00631.x 12969446

[B56] Newton S. E. Munn E. A. (1999). The development of vaccines against gastrointestinal nematode parasites, particularly *Haemonchus contortus*. *Parasitol. Today* 15 116–122. 10.1016/S0169-4758(99)01399-X 10322325

[B57] Ni L. P. Zhen Y. S. Wu S. Q. Liu H. J. Wang J. J. Hong W. (2007). A comparative analysis of the development status of the goat industry in China and the world. *Anim. Husb.* 6 30–34.

[B58] Nisbet A. J. Meeusen E. N. Gonzalez J. F. Piedrafita D. M. (2016). Immunity to *Haemonchus contortus* and vaccine development. *Adv. Parasitol.* 93 353–396. 10.1016/bs.apar.2016.02.011 27238008

[B59] Papadopoulos E. Gallidis E. Ptochos S. (2012). Anthelmintic resistance in sheep in Europe: a selected review. *Vet. Parasitol.* 189 85–88. 10.1016/j.vetpar.2012.03.036 22503039

[B60] Prichard R. (2001). Genetic variability following selection of *Haemonchus contortus* with anthelmintics. *Trends Parasitol.* 17 445–453. 10.1016/S1471-4922(01)01983-3 11530357

[B61] Qin J. H. Feng X. L. Zhao Y. L. Zhang H. Y. Cui P. Bao Y. Z. (2003). Seasonal-dynamic study of predominant wireworm of digestive canal of sheep in Bashang Altiplano Area, Hebei province. *Chin. J. Anim. Infect. Dis.* 11 12–14.

[B62] Redman E. Sargison N. Whitelaw F. Jackson F. Morrison A. Bartley D. J. (2012). Introgression of ivermectin resistance genes into a susceptible *Haemonchus contortus* strain by multiple backcrossing. *PLoS Pathog.* 8:e1002534 10.1371/journal.ppat.1002534 PMC3280990 22359506

[B63] Roeber F. Jex A. R. Gasser R. B. (2013a). Impact of gastrointestinal parasitic nematodes of sheep, and the role of advanced molecular tools for exploring epidemiology and drug resistance - an Australian perspective. *Parasit. Vectors* 6:153 10.1186/1756-3305-6-153 PMC3679956 23711194

[B64] Roeber F. Jex A. R. Gasser R. B. (2013b). Advances in the diagnosis of key gastrointestinal nematode infections of livestock, with an emphasis on small ruminants. *Biotechnol. Adv.* 31 1135–1152. 10.1016/j.biotechadv.2013.01.008 23376340 PMC7126997

[B65] Rufener L. Kaminsky R. Maser P. (2009). *In vitro* selection of *Haemonchus contortus* for benzimidazole resistance reveals a mutation at amino acid 198 of beta-tubulin. *Mol. Biochem. Parasitol.* 168 120–122. 10.1016/j.molbiopara.2009.07.002 19616042

[B66] Saddiqi H. A. Jabbar A. Sarwar M. Iqbal Z. Muhammad G. Nisa M. (2011). Small ruminant resistance against gastrointestinal nematodes: a case of *Haemonchus contortus*. *Parasitol. Res.* 109 1483–1500. 10.1007/s00436-011-2576-0 21842390

[B67] Silvestre A. Cabaret J. (2002). Mutation in position 167 of isotype 1 beta-tubulin gene of Trichostrongylid nematodes: role in benzimidazole resistance? *Mol. Biochem. Parasitol.* 120 297–300. 10.1016/S0166-6851(01)00455-8 11897135

[B68] Smith T. S. Graham M. Munn E. A. Newton S. E. Knox D. P. Coadwell W. J. (1997). Cloning and characterization of a microsomal aminopeptidase from the intestine of the nematode *Haemonchus contortus.* *Biochim. Biophys. Acta* 1338 295–306. 10.1016/S0167-4838(96)00204-X 9128148

[B69] Smith T. S. Munn E. A. Graham M. Tavernor A. S. Greenwood C. A. (1993). Purification and evaluation of the integral membrane protein H11 as a protective antigen against *Haemonchus contortus*. *Int. J. Parasitol.* 23 271–280. 10.1016/0020-7519(93)90150-W 8496010

[B70] Song M. X. Ma G. P. Han C. X. Zhu J. W. Yuan J. Q. Zhu Y. M. (2007). Observation of seasonal incidence of egg output and adult worm burden of gastro-intestinal nematodes in sheep in Songhuajiang area in Heilongjiang Province of China. *Vet. Sci. China* 37 274–276.

[B71] Sun W. Song X. Yan R. Xu L. Li X. (2011). Vaccination of goats with a glutathione peroxidase DNA vaccine induced partial protection against *Haemonchus contortus* infection. *Vet. Parasitol.* 182 239–247. 10.1016/j.vetpar.2011.05.024 21680097

[B72] Sun Y. M. Yan R. F. Muleke C. I. Zhao G. W. Xu L. X. Li X. R. (2007a). Recombinant galectins of *Haemonchus contortus* parasite induces apoptosis in the peripheral blood lymphocytes of goat. *Int. J. Pept. Res. Ther.* 13 387–392. 10.1007/s10989-006-9045-0

[B73] Sun Y. M. Yan R. F. Muleke C. I. Zhao G. W. Xu L. X. Li X. R. (2007b). Vaccination of goats with recombinant galectin antigen induces partial protection against *Haemonchus contortus* infection. *Parasite Immunol.* 29 319–326. 10.1111/j.1365-3024.2007.00949.x 17518950

[B74] Tak I. R. Dar J. S. Dar S. A. Ganai B. A. Chishti M. Z. Ahmad F. (2015). A comparative analysis of various antigenic proteins found in *Haemonchus contortus*–a review. *Mol. Biol.* 49 883–890. 10.7868/s002689841506021x 26710767

[B75] Tang D. C. DeVit M. Johnston S. A. (1992). Genetic immunization is a simple method for eliciting an immune response. *Nature* 356 152–154. 10.1038/356152a0 1545867

[B76] Taylor M. A. Coop R. L. Wall R. L. (2016). *Veterinary Parasitology* 4rd Edn. Ames, IA: Blackwell Publishing Ltd.

[B77] Wang C. R. Ma G. F. Wang Z. F. Liu X. L. Liu W. Qu B. K. (2005). Etiological investigation and integrated control technology of sheep parasitic diseases in Daqing City. *Heilongjiang Anim. Sci. Vet. Med.* 2005 48–51.

[B78] Wang C. R. Qiu J. H. Zhu X. Q. Han X. H. Ni H. B. Zhao J. P. (2006). Survey of helminths in adult sheep in Heilongjiang Province, People’s Republic of China. *Vet. Parasitol.* 140 378–382. 10.1016/j.vetpar.2006.04.008 16713098

[B79] Wang Q. Shen J. Gu J. C. (2000). Detection of resistance of stronglid nematode to albendazole on a sheep farm by egg hatch test. *Anim. Husb. Vet. Med.* 32 5–6.

[B80] Wang W. Wang S. Zhang H. Yuan C. Yan R. Song X. (2014a). Galectin Hco-gal-m from *Haemonchus contortus* modulates goat monocytes and T cell function in different patterns. *Parasit. Vectors* 7:342 10.1186/1756-3305-7-342 PMC4117971 25056558

[B81] Wang W. Yuan C. Wang S. Song X. Xu L. Yan R. (2014b). Transcriptional and proteomic analysis reveal recombinant galectins of *Haemonchus contortus* down-regulated functions of goat PBMC and modulation of several signaling cascades *in vitro*. *J. Proteomics* 98 123–137. 10.1016/j.jprot.2013.12.017 24401599

[B82] Wang Y. K. Cang N. G. Hou H. M. Kang M. (2014c). Investigation of sheep parasite in Kuze County. *Hubei J. Anim. Vet. Sci.* 35 8–11.

[B83] Wei C. Y. Sun L. J. Lin Q. (2013). Preliminary investigation on prevalence of parasites in Sheep in Yuyang District Yulin City. *J. Anim. Sci. Vet. Med.* 32 1–3.

[B84] Xia Y. G. Wang Q. (2010). Egg hatch assay for detection of levamisole resistance in *Haemonchus contortus* in goat. *Anim. Husb. Vet. Med.* 42 87–89.

[B85] Xin Y. C. (2010). Survey on parasite flora of sheep in Maying Town of Minhe County of Qinghai Province. *Anim. Husb. Feed Sci.* 31 168–169.

[B86] Yan B. Guo X. Zhou Q. Yang Y. Chen X. Sun W. (2014). *Hc-fau*, a novel gene regulating diapause in the nematode parasite *Haemonchus contortus*. *Int. J. Parasitol.* 44 775–786. 10.1016/j.ijpara.2014.05.011 25058511

[B87] Yan R. Li X. (2005). Expression of recombinant H11 of *Haemonchus contortus* in *Pichia pastoris*. *J. Nanjing Agric. Univ.* 28 85–89.

[B88] Yan R. Li X. (2006). Cloning, expression of aminopeptidase gene and analysis of recombinant protein activity in *Haemonchus contortus*. *Chin. J. Vet. Sci.* 26 151–154.

[B89] Yan R. Sun W. Song X. Xu L. Li X. (2013). Vaccination of goats with DNA vaccine encoding Dim-1 induced partial protection against *Haemonchus contortus*: a preliminary experimental study. *Res. Vet. Sci.* 95 189–199. 10.1016/j.rvsc.2013.02.020 23545480

[B90] Yan R. Wang J. Xu L. Song X. Li X. (2014). DNA vaccine encoding *Haemonchus contortus* actin induces partial protection in goats. *Acta Parasitol.* 59 698–709. 10.2478/s11686-014-0298-z 25236283

[B91] Yan R. Xu L. Li X. (2007). Immunity of recombinant *Haemonchus contortus* H11 in goats. *Chin. J. Vet. Sci.* 27 842–844.

[B92] Yang S. Q. Shi C. Q. Jian W. X. Yang G. Y. Huang S. X. (2014). Investigation of cattle and sheep parasites in Yuping County. *Guizhou Anim. Sci. Vet. Med.* 38 29–33.

[B93] Yang S. W. (2005). Parasitic disease and control strategy of goats in Youyan. *Stock Breed. Market* 8 23–25.

[B94] Yang X. Gasser R. B. Fang R. Zeng J. Zhu K. Qi M. (2016). First survey of parasitic helminths of goats along the Han River in Hubei Province, China. *Acta Parasitol.* 61 602–606. 10.1515/ap-2016-0080 27447226

[B95] Yang X. Qi M. W. Zhang Z. Z. Gao C. Wang C. Q. Lei W. Q. (2017). Development and Evaluation of a loop-mediated isothermal amplification (lamp) assay for the detection of *Haemonchus contortus* in goat fecal samples. *J. Parasitol.* 103 161–167. 10.1645/16-157 28098507

[B96] Yang Y. Ma Y. Chen X. Guo X. Yan B. Du A. (2015). Screening and analysis of *Hc-ubq* and *Hc-gst* related to desiccation survival of infective *Haemonchus contortus* larvae. *Vet. Parasitol.* 210 179–185. 10.1016/j.vetpar.2015.03.020 25913452

[B97] Yang Z. Y. Wang Z. Y. (1984). Investigation report of cattle and sheep parasites in Baoding area. *J. Agric. Univ. Hebei* 7 141–145.

[B98] Yin F. Gasser R. B. Li F. Bao M. Huang W. Zou F. (2013). Genetic variability within and among *Haemonchus contortus* isolates from goats and sheep in China. *Parasit. Vectors* 6:279 10.1186/1756-3305-6-279 PMC3852563 24499637

[B99] Yin F. Gasser R. B. Li F. Bao M. Huang W. Zou F. (2016). Population structure of *Haemonchus contortus* from seven geographical regions in China, determined on the basis of microsatellite markers. *Parasit. Vectors* 9:586 10.1186/s13071-016-1864-z PMC5111246 27846862

[B100] Yuan C. Zhang H. Wang W. Li Y. Yan R. Xu L. (2015). Transmembrane protein 63A is a partner protein of *Haemonchus contortus* galectin in the regulation of goat peripheral blood mononuclear cells. *Parasit. Vectors* 8:211 10.1186/s13071-015-0816-3 PMC4404006 25879191

[B101] Zhang H. Zhou Q. Yang Y. Chen X. Yan B. Du A. (2013). Characterization of heat shock protein 70 gene from *Haemonchus contortus* and its expression and promoter analysis in *Caenorhabditis elegans*. *Parasitology* 140 683–694. 10.1017/s0031182012002168 23360558

[B102] Zhang Z. Gasser R. B. Yang X. Yin F. Zhao G. Bao M. (2016). Two benzimidazole resistance-associated SNPs in the isotype-1 beta-tubulin gene predominate in *Haemonchus contortus* populations from eight regions in China. *Int. J. Parasitol. Drugs Drug Resist.* 6 199–206. 10.1016/j.ijpddr.2016.10.001 27760394 PMC5078572

[B103] Zhao A. P. He C. Tang J. W. Qiao D. Yang S. Li M. L. (2016). Investigation of goat parasitic infection in Nujiang Lisu Autonomous Prefecture of Yunnan Province. *Chin. J. Anim. Health Inspect.* 33 12–14.

[B104] Zhao G. Yan R. Muleke C. I. Sun Y. Xu L. Li X. (2012). Vaccination of goats with DNA vaccines encoding H11 and IL-2 induces partial protection against *Haemonchus contortus* infection. *Vet. J.* 191 94–100. 10.1016/j.tvjl.2010.12.023 21330170

[B105] Zhao J. S. Pu W. B. Zhan Z. Y. Yue C. (2010). Anthelmintic resistance of nematodes in Trichostrongylidae to benzimidazoles. *Chin. Vet. Sci.* 40 528–531. 10.1007/BF02896961

[B106] Zhou Q. J. Yang Y. Guo X. L. Duan L. J. Chen X. Q. Yan B. L. (2014). Expression of *Caenorhabditis elegans*-expressed Trans-HPS, partial aminopeptidase H11 from *Haemonchus contortus*. *Exp. Parasitol.* 145 87–98. 10.1016/j.exppara.2014.08.005 25128369

[B107] Zhou Q. J. Zhang H. L. Jiang X. L. Du A. F. (2010). The gene structure and promoter region of the vaccine target aminopeptidase H11 from the blood-sucking nematode parasite of ruminants, *Haemonchus contortus*. *Funct. Integr. Genomics* 10 589–601. 10.1007/s10142-010-0172-5 20437190

